# River Regulation Causes Rapid Changes in Relationships Between Floodplain Oak Growth and Environmental Variables

**DOI:** 10.3389/fpls.2019.00096

**Published:** 2019-02-05

**Authors:** Maksym Netsvetov, Yulia Prokopuk, Radosław Puchałka, Marcin Koprowski, Marcin Klisz, Maksym Romenskyy

**Affiliations:** ^1^Department of Phytoecology, Institute for Evolutionary Ecology, National Academy of Sciences of Ukraine, Kiev, Ukraine; ^2^Department of Ecology and Biogeography, Faculty of Biology and Environmental Protection, Nicolaus Copernicus University, Toruń, Poland; ^3^Department of Silviculture and Genetics, Forest Research Institute, Raszyn, Poland; ^4^Department of Life Sciences, Faculty of Natural Sciences, Imperial College London, London, United Kingdom; ^5^Department of Zoology, Stockholm University, Stockholm, Sweden

**Keywords:** *Quercus robur*, tree-ring, intra-annual ring-width, temperature, precipitation, water level, floodplain

## Abstract

The radial growth of pedunculate oak (*Quercus robur*), a species often ecologically dominating European deciduous forests, is closely tied up with local environmental variables. The oak tree-ring series usually contain a climatic and hydrologic signal that allows assessing the main drivers of tree growth in various ecosystems. Understanding the climate-growth relationship patterns in floodplains is important for providing insights into the species persistence and longevity in vulnerable riverine ecosystems experiencing human-induced hydrology alteration. Here, we use 139 years long instrumental records of local temperature, precipitation, and water levels in the Dnipro River in Kyiv to demonstrate that the implementation of river regulation has decoupled the established relationship between the radial growth of floodplain oak and local hydro-climatic conditions. Before the river flow has been altered by engineering modifications of 1965–1977, the water level in the Dnipro River was the key driver of oak radial growth, as reflected in the tree-ring width and earlywood width. The construction of two dams has altered the seasonal distribution of water level diminishing the positive effect of high water on oak growth and subsequently reversing this trend to negative, resulting from a seasonal ground water surplus. The decrease in the correlation between oak growth indices and the river’s water level in April–June was unprecedentedly rapid and clearly distinguishable among other changes in the growth-to-climate relationship. Our findings further demonstrate that trees growing in areas exposed to urban development are the most susceptible to downside effects of river regulation.

## Introduction

Riparian forests play a pivotal role in ecological processes at various scales (Naiman et al., 2005) and represent the most productive terrestrial ecosystems in the world. Yet, they are considered to be highly vulnerable, and without planned adaptation exposed to the grave consequences of climate change and human impact (Capon et al., 2013). The susceptibility of riparian ecosystems to natural disturbances often results in their degradation and alteration caused, among other factors, by hydrological modifications such as installation of dams and levees, water extraction, etc. (Stella and Bendix, 2019). River regulation sways a number of hydro-geomorphic processes (Stella et al., 2013) translating its overarching intra-system impact on growth of floodplain trees, density of forest stands, their structure and composition (Maděra and Úradnĉcek, 2001; Rodríguez-González et al., 2010; Smith et al., 2013; Gee et al., 2014; Stojanović et al., 2015; Weissbrod and Binder, 2017).

Disentangling the intricacies of long-term responses of individual trees, stands or entire ecosystems to river flow modification requires thorough retrospective investigations employing reliable proxies with at least annual precision accuracy. In temperate zones, the yearly variation of tree radial growth serves an effective tool for extracting the high-frequency environmental signal as well as event-driven disturbance information (St. George, 2010a,b; Esper et al., 2015). Although a number of woody plants compositing a particular ecosystem are potentially useful for developing the ring-width records, the species that contributes the most to the system’s structure and functions is of the key interest in retrospective ecological and climatological analyses. In European forests and woodlands, pedunculate oak (*Quercus robur* L.), a long-lived species, performs important ecosystem-wide functions (Nilsson et al., 2002) dominating forests growing in varying hydro-climatic and soil conditions (Ducousso and Bordacs, 2004; Ellenberg, 2009). Although European flooded forests are typically dominated by black alder or several co-dominant species, pedunculate oak particularly often composes riverine ecosystems in the areas exposed to short-term floods (Klimo and Hager, 2000; Didukh and Aloshkina, 2012). Unlike many other woody plants, the high ecological plasticity of this species enables a direct comparison of the climatic signal from nearby habitats varying by local conditions, e.g., floodplain sites adjacent to out of the valley areas.

The oak ring-width, intra-annual ring-width, and xylem anatomy measurements proved to be promising in assessing the growth-to-climate relationships in bog sites (Scharnweber et al., 2015; García-González and Souto-Herrero, 2017) and in areas experiencing flooding (Gričar et al., 2013; Hafner et al., 2015; Kames et al., 2016; Tumajer and Treml, 2016; Okoński, 2017), seasonal waterlogging (Rozas and García-González, 2012; Scharnweber et al., 2013) or human-induced hydrology modification (Singer et al., 2013; Gee et al., 2014; Stojanović et al., 2015; Tumajer and Treml, 2017; Zheng et al., 2017). However, only a limited number of studies have actually employed at least some of these tools to study the growth-hydrology relationships in pedunculate oak or in other ring-porous tree species (St. George and Nielsen, 2003; Kames et al., 2016; Koprowski et al., 2018). Even fewer works have looked into the impact of hydrology modifications on growth of floodplain trees and its relationships with environmental drivers (Predick et al., 2009; Ğejková and Polákova, 2012; Stella et al., 2013; Gee et al., 2014; Weissbrod and Binder, 2017).

In this study, we provide a comprehensive assessment of the effect of the implementation of river regulation in Kyiv, Ukraine, on relationships between the floodplain oak radial growth and local hydro-climatic conditions. Based on detailed, 139 years long, instrumental meteorological and water level records, we quantify the pedunculate oak intra-annual ring width chronologies for almost all sites embedded in the Dnipro River’s floodplain in Kyiv. Given a strong regional climate signal exhibited at all study sites, we dissect the original series using chronology from an unflooded reference forest, and applying an adjustment technique similar to that from Meco and Baisan (2001) to all study sites’ series, thereby isolating the floodplain-specific signal. Based on these data, we assess the following hypotheses: (1) Floodplain sites’ intra-annual ring-width chronologies share a specific climate/hydrology signal distinct from an adjacent area out of the valley; (2) Implementation of river regulation alters the relationships between floodplain oak growth and climate/hydrology; (3) Upon flood-preventing river modification, the oak growth is substantially relying on the river’s water level during spring (April–May) as it coincides with the early xylem development stage.

## Materials and Methods

### Sampling Sites

**FIGURE 1:**
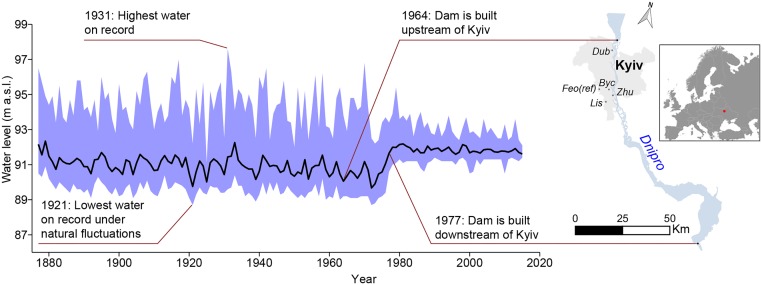
Mean annual water level fluctuations (black line) and minimum-maximum range (shading) of the Dnipro River at Kyiv over the period 1877–2015. Maps are generated in Qgis 2.14 software (https://www.qgis.org/).

Tree core sampling was undertaken at five sites (Table 1 and Figure 1) in Kyiv, Ukraine. Three study sites are located in the Dnipro River floodplain adjacent to the river stream channel: Zhukiv island (Zhu, a nature reserve), Dubysche (Dub, an oak forest), and Bychok (Byc, a reserve forest). Soil in this area is sandy alluvial and the forest floor is formed by a thick litter layer. Another study site, the Lisnyky forest botanical reserve (Lis), is part of the flooded lowland between two tributaries of the Dnipro River – the Siverka River and the Petil River. In the 1950s, the tributary rivers have been modified through drainage and ditch network maintenance that significantly depleted their flow regime. This area historically experiences seasonal inundation and backwater due to flooding events at the main stem river. The soil type here is podsolgley. Our reference site, the forest of Feofania [Feo (ref)] is located about 5 km west and 65–90 m upslope of the Dnipro River. The area does not experience seasonal floods and the groundwater level is consistently 5–20 m below the surface throughout the year. The soil in the forest is clay podsol. Byc is the only site among studied that is situated in a built-up area gradually developed since the 1950s.

**Table 1:** Sampling sites information.

Site name	Site code	Latitude (°N)	Longitude (°E)	Altitude (M)	No. of trees/cores	Earliest tree-ring	MSSL
Zhukiv	Zhu	50.339	30.588	92–94	25/60	1833	139
Dubysche	Dub	50.536	30.500	95–99	17/40	1841	156
Bychok	Byc	50.357	30.552	98–102	21/55	1839	154
Lisnyky	Lis	50.295	30.549	93–115	40/90	1866	123
Feofania	Feo (ref)	50.344	30.495	160–180	51/127	1746	180

*MSSL, mean sample segment length.*

### Tree-Ring Data

At the study sites, only visibly healthy and dominant or co-dominant oaks were selected for tree-ring sampling. Using a 5-mm-diameter increment borer, we extracted two to three cores from each tree at 1.3 m stem height. All obtained samples were glued onto the wooden supports, surfaced using blade, and scanned with a flatbed scanner (Epson Perfection V37) at a resolution of 3200 dpi. Tree ring-widths were measured to the nearest 0.01 mm resolution with AxioVision 4.9.1 software (Carl Zeiss). The cores were crossdated and merged by simple arithmetic averaging to obtain individual series. To reduce a non-climate-related signal, the individual series were detrended using a smoothing spline with a wavelength of 0.67 series duration and then standardized to produce dimensionless ring-width indices (RWI). The site-level chronologies were then built by calculating robust means for the individual series prewhitened with a best-fit first-order autoregressive model. The quality of crossdating was checked with COFECHA software (Holmes, 1983) and dplR package (Bunn, 2010) in R 3.5.1 (R Core Team, 2018). The dimensionless earlywood (EW) and latewood (LW) width chronologies, were developed from the subsets of trees that highly correlated (*r* > 0.7, *p* < 0.01) with the corresponding site-level COFECHA master-series. In LW analysis, we used its indices adjusted with respect to the known dependence of LW on EW (Meco and Baisan, 2001). Adjusted LW series were produced by fitting the linear regression of LW to EW (for correlations between intra-annual width series see Supplementary Figure 1). To pinpoint the tree growth variability inherent to floodplains (Byc, Zhu, Dub, and Lis), we applied a simple linear regression, i.e., an adjustment of RWI, EWI, and LWI (with I standing for indices) study sites’ chronologies to the corresponding reference site [Feo (ref)] chronologies. To find a year of a possible change in the mean growth rate due to river regulation, we applied the change point analysis (Killick and Eckley, 2014) to raw RW, EW, and LW chronologies. In all further analyses, we used the adjusted series from the flooded sites.

### Climate and Water Level Data

The Dnipro River has been modified in the 1920–1970s at its Ukraine stretch to generate hydroelectricity, provide water supply for agricultural needs, and mitigate extreme flooding events (Vyshnevsky, 2005). In Kyiv, the river flow modification system has included installation of dams and levees upstream (1964) and downstream (1977) of the city, which substantially moderated both the overbank and low water. We obtained data on climate and hydrology in Kyiv from the Central Geophysical Observatory (CGO, 50.40 N, 30.34′ E, 166 m a.s.l.), Kyiv, Ukraine. The available data spanned the period from 1877 through 2015. Based on the times of the main engineering modifications of the Dnipro River (1964 and 1977), we have identified two distinct study periods within our continuous (1877–2015) climate and hydrology records: before river regulation (1877–1964, first period) and during river regulation (1978–2015, second period).

**FIGURE 2:**
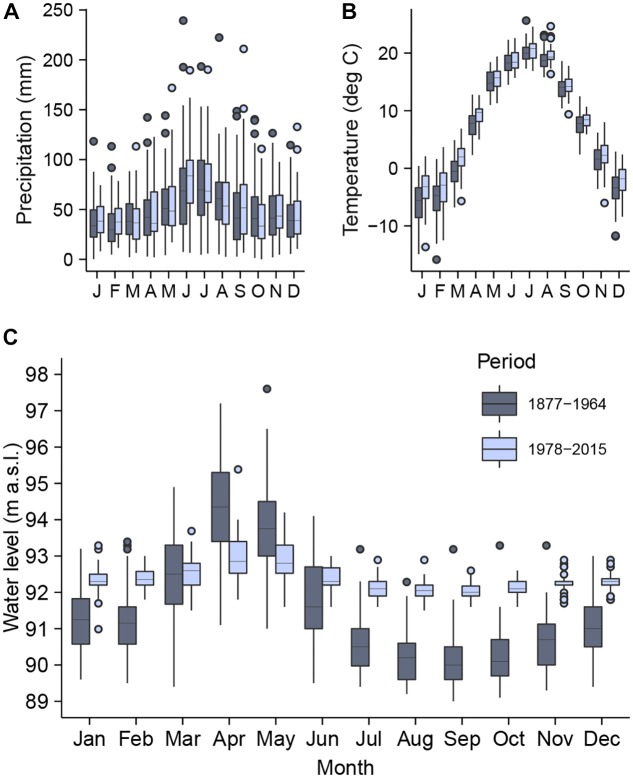
The distribution of monthly total precipitation **(A)**, mean air temperature **(B)**, and maximum water level **(C)** of the Dnipro River in Kyiv for the periods before (1877–1964) and after (1978–2015) the onset of the river regulation. In the box-and-whisker plots, the lower and upper hinges indicate the 25th and 75th percentiles, the horizontal lines denote the median values, the whiskers extend from the hinges to the largest and smallest values within the 1.5 inter-quartile range, and the points indicate outliers.

According to hydrology data, the largest floods in Kyiv (Figure 1) occurred in springs of 1877, 1908, 1917, 1931, 1970 (first period), and 1979 (second period) and have in general been attributed to rapid temperature changes and high amounts of precipitation in the river’s catchment area above the city. The lowest water level, under natural fluctuations, was recorded in November of 1921 (88.74 m a.s.l.). Following establishment of the first dam below Kyiv in 1964, the Dnipro River’s water level has decreased significantly, falling to its historical minimum (88.58 m a.s.l.) in the summer-autumn of 1972. In December 1977, another dam above Kyiv was implemented causing Dnipro’s mean annual water level to increase by 0.85 m while lowering its annual maximum by 1.32 m. It is worth to note that the engineering modification of the Dnipro River has altered the magnitude of the water level fluctuations rather than the seasonal water level distribution (Figure 2). Since 1978, the most prominent changes in the river water level were its seasonal decrease in April–May and augmentation in July–February. The latter is likely attributed to the increasing trends in June and September precipitation as well as in autumn-winter temperatures (Netsvetov et al., 2018).

The climate data (recorded at CGO) have showed higher monthly temperatures in Kyiv during the second period as compared to the first period (*p* < 0.05, *t*-test for means) for all months except June, July, September, and November. The average monthly temperature was −5.9 and −3.7°C in January and 20.0 and 20.5°C in July for the periods of 1878–1964 and 1978–2015, respectively. The annual temperature averaged 7.2°C and 8.5°C (*p* < 0.05) during the first and second period, respectively. The average total annual precipitation increased significantly (*p* < 0.05) from 590 mm in the first period to 628 mm in the second period, although its average monthly values have not showed a significant change. The month of occurrence of maximal precipitation value has shifted from July in the first period to June in the second period. The lowest precipitation value for all years since 1978 has been consistently recorded in January (Figure 2). The change in temperature in Kyiv was recognized being in line with the overall temperature trend in the northern part of the river’s catchment area (Vyshnevsky, 2005).

### Growth-to-Hydrology and Growth-to-Climate Relationships

We assessed relationships between oak growth and environmental variables, water level and climate, using bootstrapped correlation and response functions (Fritts et al., 1971; Biondi and Waikul, 2004). To establish the growth-to-climate relationship, we used a climatic window spanning from June of the previous growing season through September of the current growing season. The water level data were processed with a climatic window extending from April to September of the current year, thereby capturing a complete oak radial growth season in Kyiv. Given the multicollinearity of climatic and hydrological variables, to identify the drivers of oak growth in the first (1878–1964) and second (1978–2015) time periods, we used the stationary response function. For those variables where the stationary response function was found to have a statistically significant (*p* < 0.05) relationship with tree growth either over one or two periods, we computed the running correlation function with a 23-year sliding interval and tested its output for spurious low-frequency modulations (Gershunov et al., 2001). The calculations were performed using the ‘treeclim’ package (Zang and Biondi, 2013) for R. We then employed the locally weighted polynomial regression, implemented as a function in the ‘SiZer’ package (Sonderegger, 2018) for R, which yields the estimated smooth function. To classify the changes in oak growth-to-driver correlations, we used the estimated derivative with confidence intervals (Fan and Gijbels, 1996; Sonderegger et al., 2009). This allowed defining the intervals with the most pronounced changes in growth-to-driver correlations as minima and maxima of the first derivative.

## Results

### Chronologies Strength and Covariance

**FIGURE 3:**
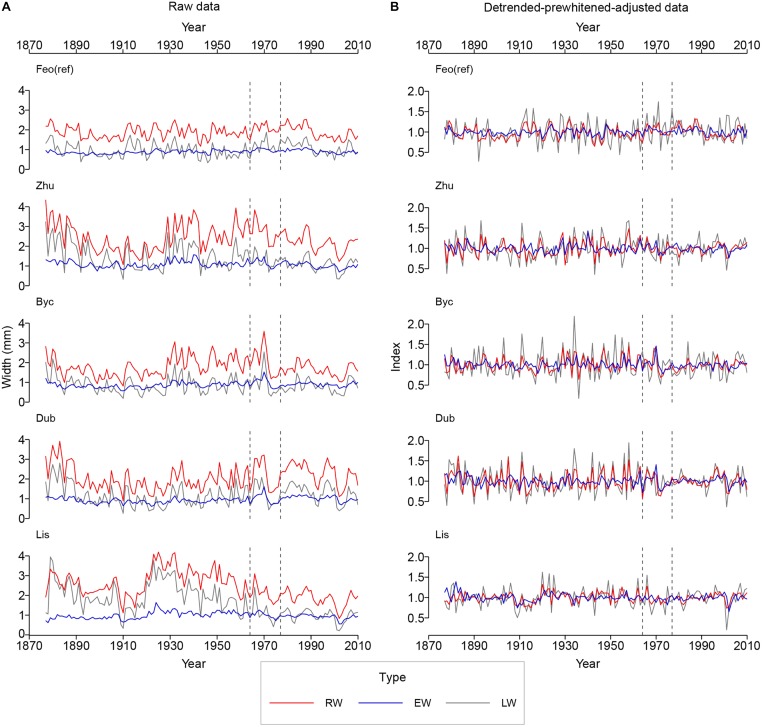
Raw **(A)** and detrended-adjusted **(B)** oak intra-annual ring-width chronologies for the reference period (1877–2015). The vertical dashed lines indicate the times of the implementation of the Dnipro River regulation systems upstream (1964) and downstream (1977) from Kyiv. The site and the chronology type codes as in Table 2.

Over the continuous period of 1877–2015, the mean intra-annual tree ring-width in the three measurement categories (radial width – RW, earlywood portion – EW, and latewood portion – LW) was higher for the floodplain sites, excluding Byc (Table 2 and Figure 3), as compared to the reference site Feo (ref). For all study sites, the change point analysis has not detected any changes in RW, EW, or LW that could be consequent of the Dnipro River regulation. The only change was identified for Lys’ RW and LW (*p* < 0.01) in 1956, well before the engineering modification of the Dnipro River stretch. The first-order autoregression coefficient (A1), a measure quantifying the persistence of chronologies, ranged from 0.46 to 0.68 for all categories. Across all sites, including Feo (ref), A1 was consistently highest in RW. Overall, A1 was lower for the reference site’s chronology than for the flooded sites’ records. The year-to-year variation, expressed by the standard deviation (SD) and mean sensitivity (MS), was considerably lower in EW than in LW and RW for all sites. The mean between-tree correlation (Reff), was in the ranges of 0.35 [Feo (ref)] – 0.43 (Dub) for RWI, 0.32 (Zhu) – 0.43 (Lis) for EWI, and 0.42 (Zhu) – 0.6 (Byc and Lis) for LWI. The expressed population signal (EPS) over the entire period for all measurement categories was greater than 0.85, suggesting a sufficient confidence level in all chronologies (Table 2).

**Table 2:** The chronologies descriptive statistics and loadings in the principal component analysis.

Site code	Type	No. of trees	EPS	Reff	Width (mm)		MS		A1	Loadings			
										Raw		Adj	
								
					Mean	*SD*	Raw	Adj		PC1	PC2	PC1	PC2
Feo (ref)	RW	51	0.95	0.35	2.1	0.76	0.2	–	0.64	−0.15	−0.05	−0.02	0.18
Zhu	RW	25	0.92	0.4	2.7	1.27	0.2	0.19	0.66	−0.43	−0.21	−0.29	−0.14
Dub	RW	17	0.93	0.47	2.2	0.98	0.25	0.23	0.61	−0.32	−0.40	−0.30	−0.42
Byc	RW	21	0.93	0.43	2	0.87	0.21	0.18	0.66	−0.28	−0.14	−0.27	−0.03
Lis	RW	40	0.96	0.43	2.4	1.21	0.15	0.12	0.75	−0.37	0.50	−0.15	−0.20
Feo (ref)	EW	12	0.86	0.38	0.9	0.19	0.13	–	0.46	−0.02	−0.02	−0.01	0.01
Zhu	EW	15	0.85	0.32	1.1	0.33	0.12	0.1	0.6	−0.09	−0.02	−0.07	−0.12
Dub	EW	13	0.86	0.34	0.9	0.27	0.12	0.11	0.54	−0.05	−0.10	−0.05	−0.19
Byc	EW	9	0.83	0.36	0.8	0.23	0.12	0.1	0.56	−0.06	−0.05	−0.07	−0.13
Lis	EW	10	0.86	0.43	1	0.27	0.1	0.09	0.58	−0.05	0.07	−0.03	−0.19
Feo (ref)	LW	12	0.92	0.53	1.1	0.6	0.35	0.35	0.53	−0.13	−0.07	0.02	0.53
Zhu	LW	15	0.9	0.42	1.5	1.01	0.35	0.32	0.58	−0.38	−0.11	−0.44	0.11
Dub	LW	13	0.92	0.5	1.3	0.86	0.4	0.39	0.52	−0.27	−0.32	−0.47	−0.16
Byc	LW	9	0.93	0.6	1.1	0.75	0.4	0.39	0.59	−0.23	0.16	−0.47	0.56
Lis	LW	10	0.93	0.6	1.6	1.11	0.26	0.24	0.68	−0.42	−0.60	−0.28	0.03

*The reference interval is 1877–2015. RW, EW, and LW stand for the radial width of a ring, the earlywood part and the latewood part, respectively; EPS is the expressed population signal; Reff is the mean correlation between trees; SD is the standard deviation; MS is the mean sensitivity; A1 denotes the first order autocorrelation; PC1 and PC2 are the first two principal components; raw and adj denote raw and adjusted series, respectively.*

**FIGURE 4:**
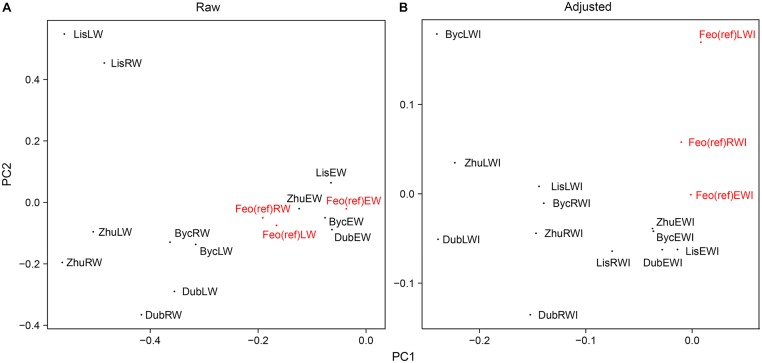
The effect of the chronologies adjustment approach on PCA results. **(A)** PCA scatter plot for raw individual series. **(B)** PCA scatter plot for detrended-prewhitened-adjusted series. Reference chronologies are in red.

Figure 4 presents results of principal component analysis (PCA). The correlation between series from the Dnipro’s floodplain and from the reference site was positive for all same-category raw chronologies (see also Supplementary Figure 1), implying that a common regional climatic signal was intrinsic to the series from all sites. The first principal component PC1 explained 51% of the total intra-annual ring-width variance between the sites, and the second component PC2 explained 25% of the variance. Yet, as many as first four principal components were required to extract ca. 90% of the total inertia. The ring-width and the intra-annual ring-width chronologies from all sites were effectively correlated with the first component that represented a mix of the common signal (observed at all sites) and the specific signal inherent to the floodplain sites (Figure 4A). Detrending and adjusting the series for flooded sites resulted in a slightly weaker MS and in a substantial decrease in correlation with the reference site, denoting the absence of the regional climatic signal in the adjusted data from the floodplain sites (Table 2). In the PCA performed on adjusted data (Figure 4B), PC1 represented the intra-annual ring-width variation mainly at the flooded sites, while PC2 corresponded to the reference site. PC1 and PC2 explained 36 and 15% of the total inertia in the adjusted data, respectively, and the first six principal components combined ca. 90% of the variance.

### Relationships Between Oak Growth and Environmental Variables

**FIGURE 5:**
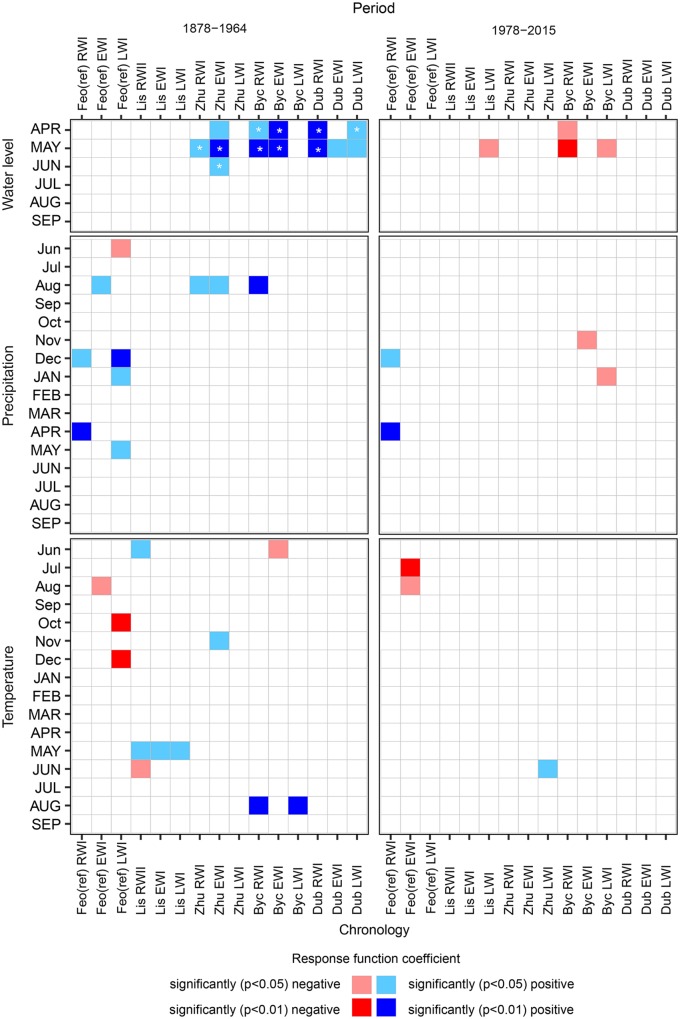
Statistically significant relationships between oak ring-width (RWI), intra-annual ring-width (EWI and LWI) chronologies and environmental variables as shown by the stationary response function analysis for the periods before (1878–1964) and after (1977–2015) the Dnipro River regulation implementation. The site and chronology type codes as in Table 2. The asterisks indicate the statistically significant changes in the growth-to-driver relationships during the entire time interval 1878–2015 (see section “Materials and Methods” for details).

The response function analysis revealed significant relationships between oak growth at the reference site [Feo (ref)] and environmental variables both for the current and preceding growing seasons (Figure 5). During the entire period of 1877–2015, Feo (ref) RWI were strongly driven by prior-December and current April precipitation and were not significantly influenced by either air temperature or Dnipro’s water level (WL). Feo (ref) EWI ring width indices were significantly tied with prior season’s August precipitation and temperature (negatively correlated), although the effect of precipitation was significant only in the first period (1878–1964). The decrease in Feo (ref) EWI over the second period (Figure 5), was also associated with the prior-July temperature. The significant relationships between Feo (ref) LWI and environmental variables spanned only for the first period. Over these years, prior-December-January and current May precipitation affected latewood growth positively, while prior-Jun precipitation, as well as October and December temperature had an overall negative effect on LWI.

The response function analysis applied to flooded sites’ data admitted numerous significant coefficients in oak grow-to-environment relationships during the first period (Figure 5). The Dnipro River’s WL, under natural fluctuations, was the main driver for floodplain oaks growth, except in Lis, which is adjacent to Dnipro’s tributaries. April WL had a significant effect on Zhu EWI, Byc RWI and EWI, Dub RWI and LWI. May WL affected both RWI and EWI growth at all floodplain sites, and also LWI in Dub. June WL had a significant influence only on Zhu LW. In the second period, upon implementation of both the upstream and downstream river regulation systems, the Dnipro’s WL effect on floodplain oak growth has diminished and subsequently reversed, i.e., in April for Byc RWI, and in May for Byc RWI and LWI. High WL in May has also become superfluous for latewood formation in Lis.

In the first period, precipitation had a positive overall effect on oak growth in the flooded sites, limited to Zhu RWI, LWI, and Byc RWI in August. More relationships were found for temperature, e.g., positive effect on Lis RWI, EWI, LWI for current May, on Byc RWI and LWI for current August, on Zhu EWI for prior-November and the negative effect on Byc EWI for prior-June. After 1977, only prior-November and current January precipitation drove Byc earlywood and latewood formation, while Zhu latewood growth was controlled by current June temperature.

**FIGURE 6:**
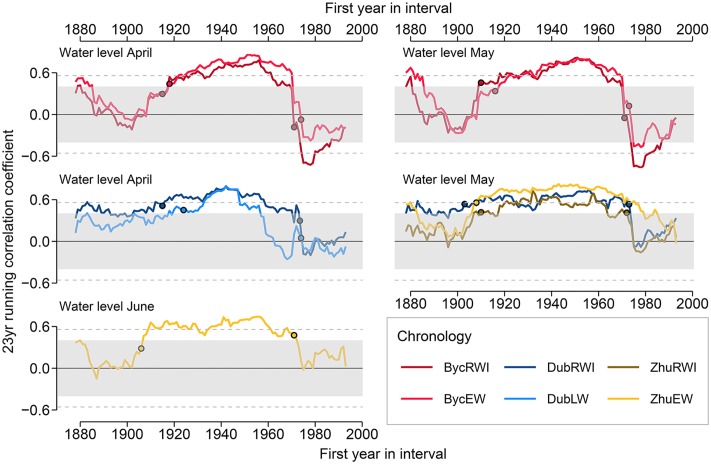
23-year running correlations between ring width and intra-annual ring-width chronologies at the Dnipro’s floodplain and the river water level. The circles indicate the start and end points of time intervals when the most rapid changes in the running correlation coefficient occurred. The shading and the horizontal lines denote the correlation coefficient thresholds *p* < 0.05 and *p* < 0.05, respectively.

The Gershunov test on temporal stability of the running correlation function coefficients confirms significant changes in relationships between oak growth and environmental variables only for WL (Figure 6). The most rapid changes occurred twice: the running correlation coefficient increased in the period of 1903–1922 and decreased during years 1971–1973. Weak correlations in the early 20th century varied in time across different sites and the reason for this remains opaque. The more coherent fall in correlation coefficients was associated with the implementation of the Dnipro regulation systems near Kyiv between 1964 and 1977. The most prominent changes were observed at Byc for RWI and EWI, where significant positive correlations with April and May WL have rapidly reversed to significantly negative correlations within the interval offset of only 3 years. The correlations were at their lowest value throughout the whole period (1978–2000) that followed the completion of river modification constructions. Similar but less abrupt changes in correlations were also registered for Zhu and Dub.

## Discussion

In this study, we used tree ring width and intra-annual ring-width chronologies (RWI, EWI, and LWI) that were additionally adjusted on reference data allowing us to accurately extract the floodplain-specific signal. All resulted floodplain chronologies contained a strong hydrologic signal, however, the most significant correlations were obtained for RWI an EWI series (Figure 5, 6). This complements some of the findings of earlier studies, which suggested that high variability of LW series reflects a strong climatic signal (Garciá-González and Eckstein, 2003). Great variability of LW might be attributed to a wide range of factors synchronizing tree growth and, consequently, LW might trace climatic information weaker than the less variable EW and its anatomical features (Fonti and Garciá-González, 2008). Despite differences among individual pedunculate oaks in the onset of cambial activity and duration of early vessel formation (Puchałka et al., 2016, 2017), an advantage of using the EW features is a relatively short period of their growth leading to limited imprinted climatic signal. In our work, strong correlations between EWI and WL, particularly in the first period, could be related to the overlap between the high water season and the period of earlywood formation.

A remarkably rapid change in relationships between oak growth and Dnipro’s WL was observed at all flooded sites, highlighting the scale of impact of river regulation on floodplain forest growth (Figure 6). Two dams on the Dnipro River near Kyiv have altered river’s annual water level oscillation by shrinking its variance throughout the season, lowering the mean water level during April–May and elevating it in June–February. This resulted in the unexpected reversal of the positive correlation between floodplain oaks growth and April–May WL as well as in more predictable dampening of growth-to-June-WL correlation. Despite the lack of data on flooded oak growth-to-hydrology relationships in the literature, the positive correlation of oak growth with WL before river regulation is in line with the results reported for ring-porous species at floodplains sites, e.g., for *Quercus lyrata* RW (Gee et al., 2014), *Q. robur* RW (Okoński, 2017), and LW (Hafner et al., 2015), for *Fraxinus excelsior* basal area increment (Singer et al., 2013) and RW (Koprowski et al., 2018). The relatively short WL rise during the early growing period favors oaks’ growth as inundations contribute to soil saturation by water and its enrichment by nutrients. This seems to play a key role at the sites where both floods and low water occurred during the radial growth season, certainly being the case for the studied forests before river regulation. The positive correlation between oak growth and river WL is also intrinsic to floodplains where human impact on hydrology has caused a decrease in a water-table level (Stojanović et al., 2015). In contrast, a negative influence exerted on oaks by floods during early growing season under river regulation resembles a similar effect attributed to the areas liable to floods (Scharnweber et al., 2015) or prone to artificially increased soil water-table (Koval et al., 2015). The physiological consequences of root anoxia (Kozlowski, 1997; Kreuzwieser et al., 2004) and mycorrhiza death (Vasilas et al., 2004) that follow the period of prolonged water excess are among main causes of bog oaks growth depression (Sass-Klassen and Hanraets, 2006; Scharnweber et al., 2015) and seem to be also relevant to our results for the second study period.

The registered growth-climate relationships confirmed water surplus at the flooded sites in the post-regulated period, when growth (Byc EWI and LWI) became negatively correlated with precipitation. Despite the warming trend in regional climate, only June temperatures have significantly and positively affected the oak growth [Zhu LWI (Figure 5)]. The weakening of the relationship between non-adjusted RWI from Lis and temperature in May has been earlier considered attributed to the known warming trend and lowering of the evaporation demand, although no linear trend has been found for precipitation in Kyiv (Netsvetov et al., 2018). The positive correlation with temperature has been also reported in other studies of flooded areas (Hafner et al., 2015; Tumajer and Treml, 2016) and areas without water deficiency (Ğejková and Polákova, 2012; Tumajer and Treml, 2017), although neither of these works has considered the effect of hydrology modification.

Generally, the modification of river flow is known to affect floodplain ecosystems through alteration of natural disturbances (Nilsson and Berggren, 2000), e.g., flooding. Our findings coupled with earlier data suggest multiple pathways to changes in the growth-to-hydrology/climate relationships after river regulation. The growth responses observed in a single valley (Stella et al., 2013) or even at a single site (Scharnweber et al., 2015; Tumajer and Treml, 2017) may differ significantly, emphasizing the crucial importance of microsite conditions for the trees’ ability to withstand artificial hydrology changes. In the Dnipro’s valley in Kyiv, all chronologies shared a similar hydrological signal but differed in the extent of changes in correlations following the damming of the river. The oak stands at Lis and Byc were found to be the most sensitive, demonstrating a strong negative LW-WL or RWI-WL relationships (Figure 5). These sites experienced hydrology alteration caused by developments in their immediate neighborhood (Byc): a levee construction along the Dnipro’s coast (Lis) and modifications of tributaries and intermittent streams (at both sites). Such factors likely impact tree growth, influencing the levels of transient reservoirs and, thus, soil-water availability in the root zone. This bias, however, cannot be reduced in our study as there are no data available on the local groundwater level. Our previous study has demonstrated that the last decades’ hydrology alteration has caused Lis oaks to suffer from both yearly growing season inundations and late-summer low-water (Netsvetov et al., 2018). The oscillation in the water table can exacerbate a severe drought impact as the EW-vessel size and density are governed by soil saturation in water during xylogenesis (Copini et al., 2016) and may became unfit to low water availability later in the season (Tumajer and Treml, 2016).

Our results show that floodplain oaks’ RWI, EWI, and LWI adjusted on the reference site’s data contain a sufficient hydrologic signal allowing us to discriminate between pre- and post-regulation periods in the river management. The rate and strength of the oak intra-annual ring-width response to river regulation varies among different study sites and is highest in the areas that have suffered from human impact on local hydrology. Though our findings show that the signal from pedunculate oak growth is an effective tool in assessing the impact of hydrological modification on riparian trees, to gain additional insight into the floodplain forests’ vulnerability, future researches should also consider the inherent site- and microsite-based variation in environmental conditions and ideally employ data on growth of multiple dominant tree species.

## Data Availability

The datasets supporting the conclusion of this study are available from the corresponding author upon request.

## Author Contributions

YP, MN, and MR contributed to conception and design of the study. YP organized the datasets. YP and MN performed the statistical analysis. MN produced the figures. MN, YP, RP, MKl, and MKo drafted the first version of the manuscript. MR, MN, and YP revised and finalized the manuscript with input from RP, MKo, and MKl. All authors read and approved the final version of the article.

## Conflict of Interest Statement

The authors declare that the research was conducted in the absence of any commercial or financial relationships that could be construed as a potential conflict of interest.
