# Supplementary material for: River Regulation Causes Rapid Changes in Relationships Between Floodplain Oak Growth and Environmental Variables
Source: Front Plant Sci. 2019 Feb 5;10:96. doi: 10.3389/fpls.2019.00096 (PMC6370973; doi:10.3389/fpls.2019.00096)
Supplement: Supplementary file 1 [file Data_Sheet_1.docx]

Supplementary Material

River Regulation Causes Rapid Changes in Relationships Between Floodplain Oak Growth and Environmental Variables

Maksym Netsvetov*, Yulia Prokopuk, Radosław Puchałka, Marcin Koprowski, Marcin Klisz, Maksym Romenskyy

*** Correspondence:** Dr. Maksym Netsvetov: disfleur76@live.fr


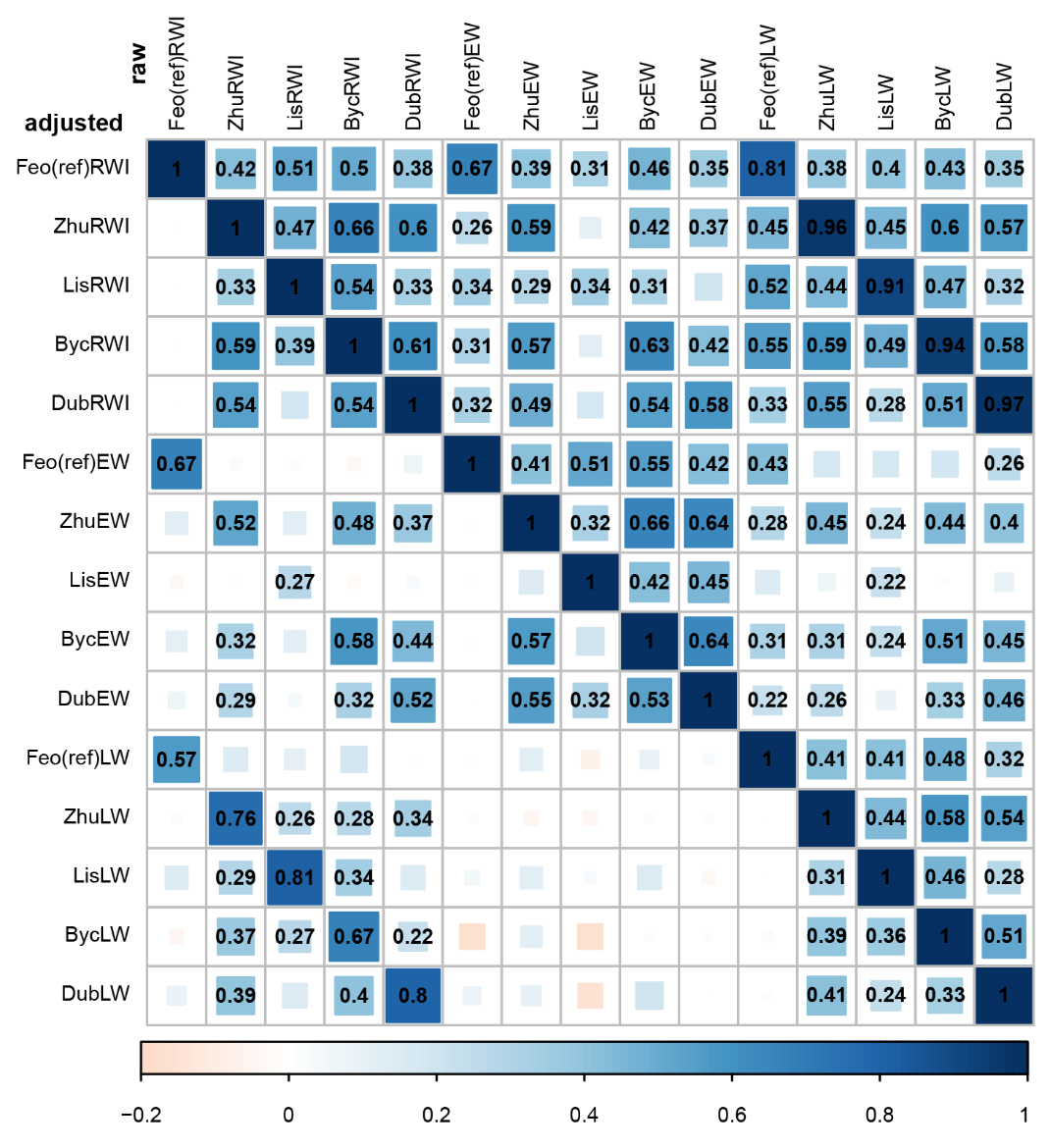


**Supplementary Figure 1.** Pearson’s correlation coefficients between chronologies using raw data (upper triangle) and data after detrending, prewhitening and adjusting (lower triangle). Only statistically significant (p<0.01) values are presented.
